# Phrenologic

**Published:** 1871-01

**Authors:** Ben. H. Pratt


### PHRENOLOGIC.

BEN. H. PRATT, A. M.

The sublime science of Phrenology, opening as it does the ponderous gates of fate, and ushering us into the mysteries of what the balance of our life-career must assuredly be, awes us. We have been skeptical upon the subject of Phrenology. We think we have met others slightly tinctured with infidel views upon this question. But we've experienced a change.—Retractions, however, are mortifying, so we don't purpose recanting what we may have spoken or written of a derogatory nature upon this important theme. Still, we don't mind making a clean breast of our conversion, and confiding to BISTOURY the manner in which it came about.

Some three years since, in the great city of New York, a gentleman of infinitely practical views upon the subject of general education, and possessing withal a deep sense of something lacking in the manner of interesting youth, started, in connection with a business college, a monthly journal, devoted to science, art, literature, education, morals, religion and Packard's Business College. S. S. Packard was a self-made man—one who had risen from the people and knew the wants of the masses. Having run the gauntlet of many professions, businesses, occupations, avocations and callings, he was of course competent to judge which best fitted him and which he best fitted. His journal was "dead set" against romances of any kind, and upheld the sensational, pure and simple. The "Wickedest Man" article achieved a noteriety for the periodical and thenceforward its star was in the ascendant. Attracted by the new and peculiar field in which this magazine purposed to labor, and by the very reasonable subscription price, we secured it from the first number, and for two years was loud in praise of its ability and fearlessness. It owned a freshness of which no other work of the kind could boast, and was effecting a great good in a department of literature yet untried, and finding its way into paths which no previous publication had ever trodden. The prospectus of '68 and that of '69 were out-done by that of '70, and we chuckled over the many good things in store for us in the way of choice literary tid-bits for the coming year.—But all at once, without one word of warning, came to our address—not Packard's welcome monthly, but a new covered, new colored, new titled, strange looking, bust-decked hybrid, which we found, upon investigating the contents and apologetic "words to the reader," to be a consolidation of our pet journal with a decaying publication of phrenological proclivities, purporting to have been published by one Wells of New York. We were not happy over the union —were first mad, then chagrined, then hurt.—Victims of a swindle which we could scarcely believe S. S. Packard could connive at, we concluded to stop our paid-for journal and put no more trust in small publishers. Upon closer examination of the very prolix and learnedly wrought dedication, however, which, as nearly as may be remembered, devoted the magazine to the "Ethnological, Physiological, Phrenological, Physiognomical, Psychological, Educational, Artistic, Scientific, Literary, Reformative, Improvative, Elevative, &c., Interests of Society, Physically, Morally, Mentally and Spiritually," we concluded that a periodical of such weight it were folly to attempt to stop; hence it has been coming ever since, and it does seem as though that subscription never would expire.

How fortunate we were in not stopping that subscription! Now, you can understand how we became such an ardent believer in, and advocate of Phrenology, Psych—and all the sublime ologies. We never before realized the force of the ancient adage, "a little learning is a dangerous thing." The little we have gleaned from removing the wrapper of said journal monthly and chucking both into the waste paper basket after glancing at the cover, has set us to ogling strangers upon the street—our friend who drops in for a chat—our butcher—our baker—our pantaloon maker—our debtors—our creditors—everybody. We read them all. Little they wot of how much of their past, present and future history we are master of—how well we know of that little matrimonial scene between Mr. and Mrs. A., this morning; and how Mr. B. is going to elope with Mrs. C. next Wednesday week at 2 A. M., precisely; and how Mr. D. is momentarily dreading the denouement of the coming statement to his creditors of his financial condition. How clearly stand out the signs of character upon " the human face divine." How true to the letter do we mentally prognosticate the fate of all we meet. Taking a firm and decisive stand upon the principles of Physiognomy, based on Ethnology, so thoroughly systematized, perspicuously explained, so transparently illustrated, so aptly applied in the ologic journal in question, one cannot go astray. To us there is a world of wealth (no cash value though) in the form of a face—in the slant of an eye—in the curve of a mouth—in the twist of a nose—in the peak of a chin—in the wag of a jaw—in the angle of a tooth—in the curve of a cheek—in the slant of a forehead—in the kink of a hair—in the flow of a beard—in the curve of a neck— in the size and shape of an ear—in the mould of a hand—in the spread of a foot—in the tone of a voice—in the swing of a gait—in the ring of a laugh—in the stroke of a pen—in the wrinkle of a palm—in a thousand little things that people do or don't do, or can't or won't do. Great is Phrenology, and Physiognomy is its prophet. How much we all owe the enterprising Packard for getting the ears of the public before injecting them with Wells' hobby. How we should all admire the magnanimity of the former in allowing the latter to mask himself in the garb of sense and decency, and then walk into our homes confidently—in allowing the pages of a popular and excellent magazine to be filled with such eminently practical knowledge as Phrenology affords. How preeminently proper—how supremely honorable in Mr. Packard to issue a prospectus which heralded the fact that an increase in size and price of his periodical would augment the knowledge of the world proportionately, and then turn over to Mr. Wells the task of fulfilling the trust.

In all seriousness, nearly a year has elapsed since we were startled by the swindle of consolidating a good magazine with an universally conceded humbug. And although we resisted the temptation to publish our opinion thereof at the time, least the heat of anger should lead us to utter words which we should afterwards regret, eight or ten months of sober thought have not changed our opinion of this mean method of foisting a pure humbug upon an unsuspecting public. The sooner the abortion known as the Phrenological Journal and Packard's Monthly ceases to come to our address, the better, and we are not alone in our opinion. The exposition of humbuggery is the avowed province of the BISTOURY, and we make such exposition under no assumed name. We confess ourselves swindled, but hope that all who have been similarly treated will gladly bid adieu forever to a magazine and a publisher mean enough to resort to such an under-hand method of scattering abroad his worthless, nay injurious and immoral sheets. Packard still maintains a semblance of his journal tucked away into the last few pages of Wells' pamphlet, but that by no means removes the curse that blights the work and the transaction that produced it. If the thing meets the approbation of any, we have yet to find such, and we have looked for them assiduously. There possibly are still living a few fanatics upon this, as well as every other humbug—strange if there are not; but we hope, and all with whom we have talked in reference to this matter, hope that the project will die—die as it has lived for some months, a hated and despised sheet.

—A Chicago lady dropped one of her eyebrows in the chuch pew, and dreadfully freightoned a young man sitting next to her, who thought it was his moustache.

—In New York, recently, a woman fell into a fit and was sent to a hospital, where she was relieved of a set of false teeth which she had partially swallowed, and she departed convalescent.

